# A 39-year-old women with newly diagnosed ALCAPA syndrome during pregnancy

**DOI:** 10.1007/s00392-025-02597-5

**Published:** 2025-01-29

**Authors:** Lukas Stolz, Juliane J. Schneider, Magda Haum, Heidi Estner, Jörg Hausleiter, Steffen Massberg, Manuela Thienel

**Affiliations:** 1 Medizinische Klinik Und Poliklinik I, LMU Klinikum, LMU München, Marchioninistraße 15, 81337 Munich, Germany; 2 German Center for Cardiovascular Research (DZHK), Partner Site Munich Heart Alliance, Munich, Germany; 3 Medizinische Klinik Und Poliklinik IV, Nephrologisches Zentrum, LMU Klinikum, LMU München, Munich, Germany

Sirs:

A 39-year-old women presented to her outpatient cardiologist with progressive peripheral edema and moderate exertional dyspnea (New York Heart Association [NYHA] functional class II–III) in the 39th week of pregnancy (38 + 6; Gravida 1 Para 0). Transthoracic echocardiography (TTE) revealed reduced left ventricular function and an abnormal color doppler signal across the interventricular septum. The patient was immediately referred to our center for further diagnostics and treatment.

**Fig. 1:**
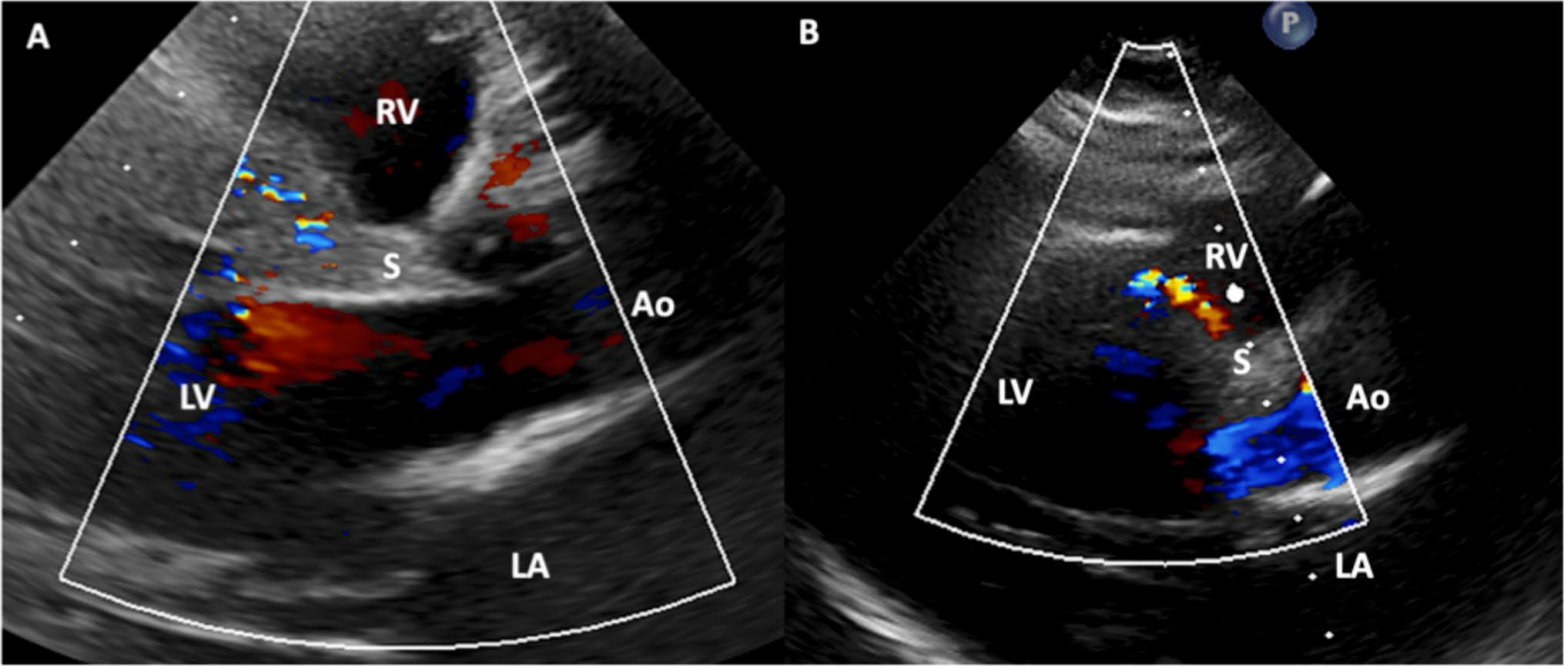
Figure 1 shows the abnormal septal TTE color doppler signal due to an excessive septal collateral system. *Ao* aorta, *LA* left atrium, *LV* left ventricle, *S* interventricular septum, *RA* right atrium, *RV* right ventricle, *TTE* transthoracic echocardiography

Besides the above-mentioned peripheral edema, physical examination was significant of a 2/6 holosystolic murmur without radiation into the carotid artery or the axilla. The electrocardiogram showed sinus tachycardia with left anterior fascicular block. TTE confirmed LV hypertrophy with a moderately reduced left ventricular ejection fraction (LVEF 40%) in the absence of severe valvular dysfunction. Even though there was abnormal color doppler signal in the region of the interventricular septum (Fig. 1), no ventricular septum defect could be detected. Laboratory workup revealed mildly elevated but stable Troponin-T levels (0.043 ng/ml; upper limit of normal 0.014 ng/ml) and elevated NT-proBNP (1279 pg/ml; upper limit of normal 237 pg/ml). Examination of the fetus (ultrasound, cardiotocography [CTG]) remained unremarkable. With the primary working hypothesis of peripartum cardiomyopathy, the indication for a caesarean section was made by interdisciplinary consensus, which was performed without significant complications. The male newborn was healthy (weight 3.2 kg, height 51.0 cm, APGAR-Score 9/10/10).

**Fig. 2:**
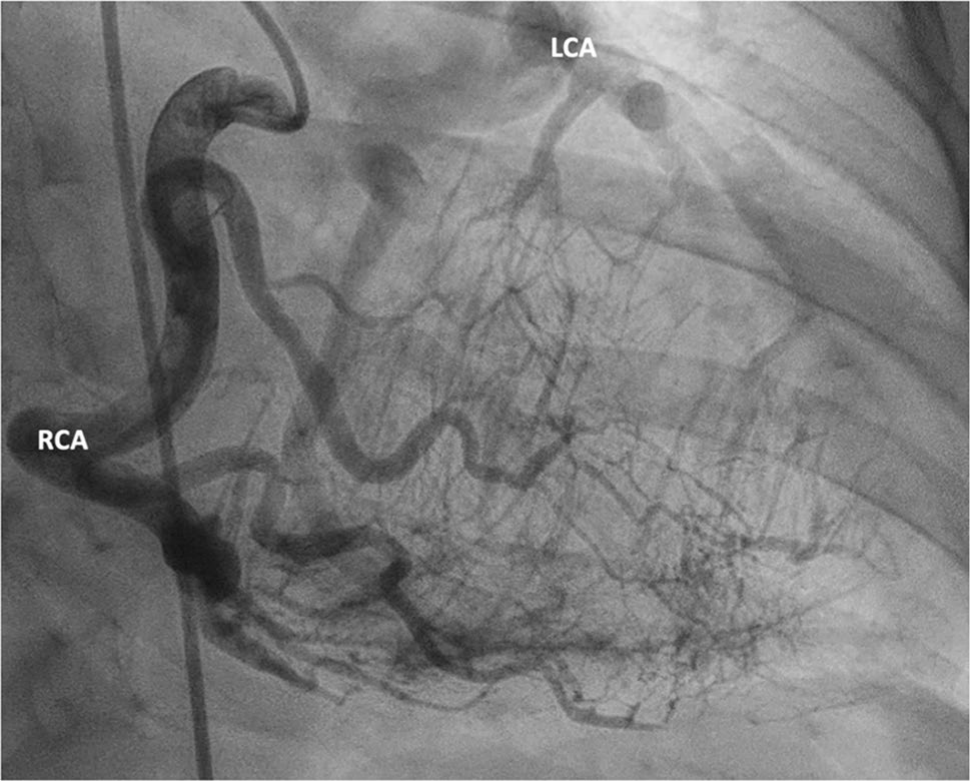
Figure 2 shows a large RCA giving rise to an extensive capillary vessel system and retrograde contrasting of the anomalous LCA from the pulmonary artery. *LCA* left coronary artery, *RCA* right coronary artery

**Fig. 3:**
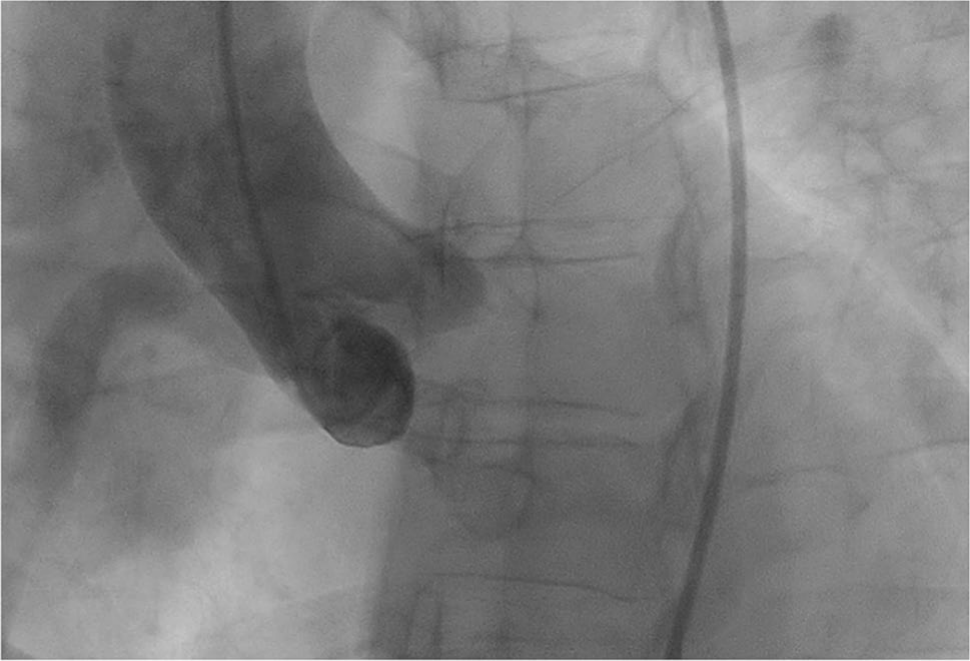
Figure 3 shows, that no LCA originating from the aorta could be identified angiographically. *LCA* left coronary artery

Against the background of a suspected peripartum cardiomyopathy an oral treatment with Bromocriptine was initiated (2.5 mg once daily for seven days). Medical treatment was supported by uptitration of guideline directed medical therapy (GDMT) for heart failure with reduced ejection fraction (HFrEF). To rule out coronary artery disease, the further work-up included coronary angiography. The latter revealed a complex coronary anomaly with retrograde contrasting of the left coronary system by an excessive network of septal branches communicating with a large dilated right coronary artery (Fig. 2). Even following aortic angiography, no left coronary artery (LCA) originating from the aorta could be identified (Fig. 3). For further classification we performed computed tomography (CT) scan of the coronary system, which classified the pathology as anomalous left coronary artery from the pulmonary artery (ALCAPA, Bland-White-Garland syndrome). Additionally, cardiac magnetic resonance imaging (CMR) was significant of a global left ventricular subendocardial late gadolinium enhancement (LGE) reflecting myocardial fibrosis and partial myocardial edema indicating newer ischemic processes. Equivalent to abnormal echocardiographic color doppler signal, an excessive capillary network was identified within the interventricular septum. Given a comparably low blood pressure after GDMT administration (about 90/70 mmHg) we reduced HF medication in favor of improved coronary perfusion. Against the background of one episode of non-sustained ventricular tachycardia (nsVT) with 34 beats in telemetric monitoring, the continued medical therapy primarily included Metoprolol.

Echocardiographic evaluation of the newborn excluded relevant coronary anomalies.

**Fig. 4:**
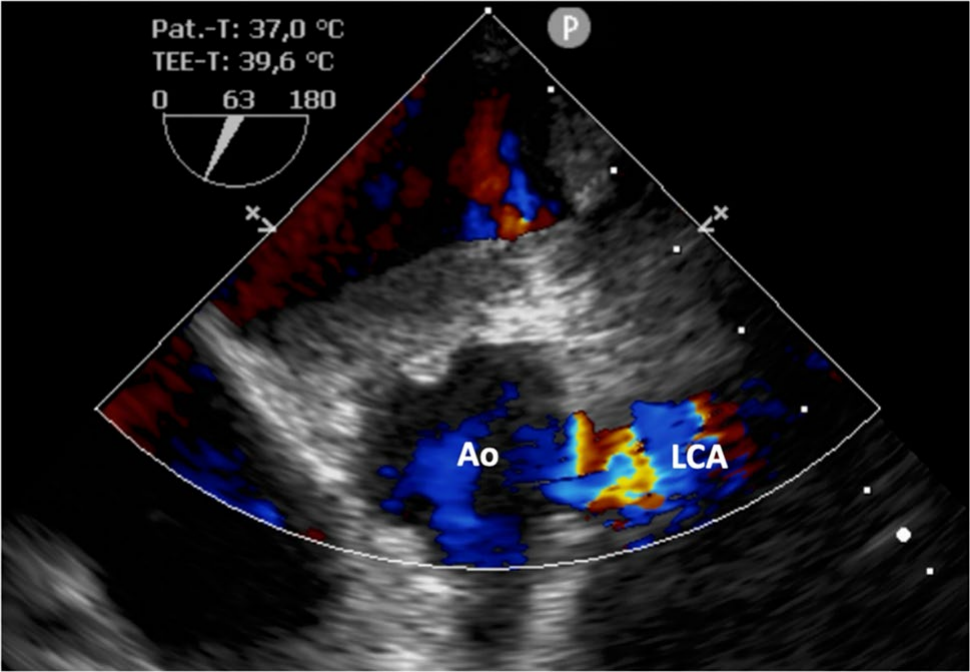
TEE imaging after connection of the LCA with the aortic root. Figure 4 shows blood flow from the aorta through the reinserted LCA. *Ao* aorta, *LCA* left coronary artery, *TEE* transesophageal imaging

The indication for surgical correction was made by consensus of an interdisciplinary heart team consisting of cardiology, cardiac surgery, pediatrics, and pediatric cardiac surgery. Gaining surgical access through a median sternotomy, an external circulation was established. The LCA was excised from its origin in the dorsal sinus of the pulmonary artery (PA) and reinserted onto the aorta. The PA was then reconstructed using a bovine pericardial patch. Intraoperative transesophageal echocardiography confirmed good perfusion of the reinserted LCA (Fig. 4). The further clinical course was unremarkable. TTE prior to discharge showed mildly reduced LV function (LVEF 43%) without relevant regional wall motion abnormalities and slightly reduced longitudinal right ventricular function (TAPSE 14 mmHg) due to surgery. The patient was discharged on the 9th postoperative day. Medical treatment at discharge included quadruple GDMT (Metoprolol 50 mg/d, Spironolactone 25 mg/d, Dapagliflozin 10 mg/d and Sacubitril/Valsartan 24/26 mg/d) and aspirin for 90 days. A clinical follow-up was scheduled three months after surgery following rehabilitation.

The present article reports the rare case of newly diagnosed ALCAPA syndrome in an adult patient. While being asymptomatic for 39 years, the patient developed clinical symptoms of LV failure unmasked by an increased hemodynamic stress in the 39th week of pregnancy.

The case report highlights the importance of a multidisciplinary approach of the Pregnancy Heart Team in the management of new onset heart failure during pregnancy as suggested in the 2018 ESC Guidelines for the management of cardiovascular diseases during pregnancy [1]. Given our patient’s unremarkable past medical history the initially most likely diagnosis was peripartum cardiomyopathy. Therefore, medical treatment included Bromocriptine early on after birth. However, potential differential diagnoses which could have worsened during pregnancy included other types of cardiomyopathy (e.g. dilated, hypertrophic, non-compaction, Takotsubo), valvular heart disease (e.g. bicuspid aortic valve, mitral valve prolapse) and congenital heart disease (e.g. ventricular septum defect) [2]. Therefore, a multimodal diagnostic approach including coronary angiography, cardiac CT and CMR was initiated.

ALCAPA itself is a rare congenital heart disease affecting about one out of 300 000 live births [3]. While the majority of patients present in infancy (85%) [4], only 15% of cases are diagnosed in adults [4]. When remaining undetected and/or untreated, ALCAPA is associated with an increased risk of sudden cardiac death due to ongoing ischemia leading to potentially fatal cardiac arrhythmias [5]. In line with this, our patient showed ventricular arrhythmias in telemetric monitoring. The fact that our patient was asymptomatic for many years is probably due to the development of extensive collateral circulation.

According to the 2018 guidelines for the management of adults with congenital heart disease, surgical correction of ALCAPA is recommended irrespective of age and symptoms [6]. Several different surgical approaches have been reported. Generally, all modern techniques aim at re-establishing a dual coronary system [7–9]. In the present case, the LCA was reimplanted onto the aortic root and a dual coronary system was re-established. At discharge, LV function already showed slight improvement. After surgery, GDMT was uptitrated and further follow-up visits will show the degree of functional LV recovery the patient might experience.

Even though being a rare condition, ALCAPA and other coronary anomalies need to be considered as differential diagnosis also in adult patients. New onset heart failure during pregnancy requires a structured diagnostic approach involving the whole Pregnancy Heart Team and gynecology in order to optimize treatment and patient outcomes.
